# The Degree of Liver Steatosis Is Associated with Abnormally High Serum Levels of Markers of Blood–Brain Barrier Dysfunction and Systemic Inflammation in Patients with Morbid Obesity

**DOI:** 10.3390/medicina62050821

**Published:** 2026-04-25

**Authors:** Gabriela Hurtado-Alvarado, Karol Iliana Ávila-Soto, Marlene Monserrat Juárez, Lucía Angélica Méndez-García, Verónica Cevallos-López, Juan Antonio Peralta-Calcaneo, Marcela Esquivel-Velázquez, Antonio González-Chávez, Julio César Zavala-Castillo, Ana Alfaro-Cruz, Jaime Héctor Gómez-Zamudio, Galileo Escobedo

**Affiliations:** 1 Departamento de Anatomía, Facultad de Medicina, Universidad Nacional Autónoma de México, Mexico City 04510, Mexico; hual.g313@gmail.com; 2 Laboratorio de Inmunometabolismo, Dirección de Investigación, Hospital General de México “Dr. Eduardo Liceaga”, Mexico City 06720, Mexico; av.karol.17@gmail.com (K.I.Á.-S.); juarezmontse192@gmail.com (M.M.J.); lucia.angelica86@gmail.com (L.A.M.-G.); veronica.cevallos1993@gmail.com (V.C.-L.); 3 Servicio de Endocrinología, Hospital General de México “Dr. Eduardo Liceaga”, Mexico City 06720, Mexico; juan_peca@hotmail.com; 4 Laboratorio de Proteómica, Dirección de Investigación, Hospital General de México “Dr. Eduardo Liceaga”, Mexico City 06720, Mexico; esquivel.marcela@gmail.com; 5 Servicio de Medicina Interna, Hospital General de México “Dr. Eduardo Liceaga”, Mexico City 06720, Mexico; antglez51@yahoo.com.mx; 6 Servicio de Cirugía General, Hospital General de México “Dr. Eduardo Liceaga”, Mexico City 06720, Mexico; jcmaximusmx@yahoo.com.mx; 7 Servicio de Anatomía Patológica, Hospital General de México “Dr. Eduardo Liceaga”, Mexico City 06720, Mexico; analfaro@yahoo.com; 8 Unidad de Investigación Médica en Bioquímica, Hospital de Especialidades “Bernardo Sepúlveda”, Centro Médico Nacional Siglo XXI, Instituto Mexicano del Seguro Social, Mexico City 06720, Mexico; jaime.gomezz@imss.gob.mx

**Keywords:** steatosis, liver biopsy, blood–brain barrier, TNF-α, IL-10, neuron-specific enolase, glial fibrillary acidic protein

## Abstract

*Background and Objectives*: The pathogenesis of liver steatosis is associated with obesity and systemic inflammation, particularly in subjects with body mass index (BMI) above 40 kg/m^2^ and altered serum levels of tumor necrosis factor alpha (TNF-α) and interleukin-10 (IL-10). Recent evidence suggests that disruption of the blood–brain barrier (BBB) may be associated with the development of steatosis, although limited data are available in humans. Thus, we assessed serum levels of neuron-specific enolase (NSE), transglutaminase 2 (TGM2), and glial fibrillary acidic protein (GFAP) as indirect markers of BBB dysfunction and examined their associations with steatosis severity, TNF-α and IL-10 in patients with morbid obesity. *Materials and Methods*: We biopsied the liver during bariatric surgery to assess steatosis by histology and serum markers by ELISA. *Results*: Most study subjects were women aged 38.7 ± 9.9 years with an average BMI of 42.3 ± 7.9 kg/m^2^ and a steatosis prevalence of 78.9%. After grading steatosis as none (*n* = 8), mild (*n* = 17), moderate (*n* = 8), or severe (*n* = 5), we found no differences in sex, age, BMI, comorbidities, or laboratory variables, including liver enzymes. One-way ANOVA showed that serum IL-10 was 4-fold less in severe steatosis than in mild steatosis (*p* = 0.038), whereas TNF-α levels increased twice in severe steatosis compared to no steatosis (*p* = 0.029). NSE and GFAP serum levels, but not TGM2, increased proportionally to steatosis stage, showing differences between severe steatosis and no steatosis (*p* = 0.012 and *p* = 0.0002, respectively). Pearson correlation coefficients showed that NSE and GFAP were significantly associated with TNF-α (*r* = 0.600 and *r* = 0.402, respectively), but not with IL-10. *Conclusions*: Steatosis severity is significantly associated with markers of BBB disruption and systemic inflammation in patients with morbid obesity, suggesting a link between the BBB and liver steatosis.

## 1. Introduction

Metabolic dysfunction-associated steatotic liver disease (MASLD) is a growing public health concern worldwide that has an intricate link to obesity and low-grade systemic inflammation (LGSI) [1,2]. MASLD encompasses a broad spectrum of liver abnormalities ranging from steatosis to steatohepatitis, fibrosis, cirrhosis, and hepatocellular carcinoma (HCC) [3]. Steatosis is the abnormal accumulation of lipids in the liver parenchyma, which marks the initial step in the MASLD spectrum, where lifestyle changes and appropriate medical interventions can reverse the condition [4]. Although hepatic lipid metabolism is associated with genetic factors, dysbiosis, dietary patterns, and LGSI, the mechanisms underlying liver steatosis remain elusive [5,6,7].

Recent evidence in elderly individuals and patients with neurodegenerative disorders suggests a possible role for blood–brain barrier (BBB) dysfunction in the development of steatosis [8,9]. Magnetic resonance imaging (MRI) studies first revealed the loss of BBB integrity during normal aging [10]. The prevalence of steatosis increases with age from 13% in adolescents to 38% and 50% in adults and seniors, respectively [11,12]. LGSI also increases with age, as shown by abnormally high serum levels of tumor necrosis factor alpha (TNF-α) and low values of interleukin-10 (IL-10) in elderly subjects with metabolic alterations or Alzheimer’s disease (AD) [13]. Moreover, patients with cognitive impairment (CI) and AD show an increased risk of developing MASLD compared to healthy individuals [14,15]. This information collectively suggests a possible link between BBB disruption, LGSI, and liver steatosis.

Several serum markers allow monitoring of neuroinflammation and provide an indirect assessment of BBB integrity, including neuron-specific enolase (NSE), transglutaminase 2 (TGM2), and glial fibrillary acidic protein (GFAP). NSE is a glycolytic enzyme found in the cytoplasm of neurons, while TGM2 is a crucial component of the neurogenic niche [16,17]. GFAP is a monomeric intermediate filament protein located in astrocytes within the central nervous system (CNS) [18]. After brain injury or acute neuroinflammation, nervous tissue releases these proteins into the cerebrospinal fluid, where they can cross into the blood circulation through an impaired BBB [18,19,20]. Thus, measuring NSE, TGM2, and GFAP serum levels allows indirect monitoring of BBB function in metabolic and neurodegenerative disorders such as obesity, type 2 diabetes (T2D), CI, or AD, where TNF-α and IL-10 also play crucial roles [21,22,23,24].

The probable association among BBB disruption, LGSI, and liver steatosis remains uncertain in patients with a high prevalence of steatosis, such as those with morbid obesity. Thus, we conducted a prospective, cross-sectional study in morbidly obese patients with varying degrees of simple steatosis, examining serum levels of markers of BBB dysfunction and LGSI, including NSE, TGM2, GFAP, TNF-α, and IL-10.

## 2. Materials and Methods

### 2.1. Patients

We conducted a prospective, cross-sectional study enrolling 38 patients of both sexes aged 18 years and above with morbid obesity. The study participants met the selection criteria for Roux-en-Y gastric bypass in the Clinic of Treatment for Patients with Obesity and Diabetes and the Surgery Department of the General Hospital of Mexico from February 2023 to September 2025. All 38 participants agreed to donate a liver specimen measuring 1.2 mm in diameter, obtained during surgery, in accordance with the Metabolic and Bariatric Surgery Accreditation and Quality Improvement Program (MBSAQIP) [25]. We excluded patients from the study if they had a prior diagnosis of endocrine disorders, infectious diseases, autoimmune disorders, or cancer, or had received immunomodulatory drug therapy within the previous 3 months. All enrolled participants signed the informed consent approved by the Ethics Committee of the General Hospital of Mexico, with registration number DI/16/UME/05/048. We conducted the study in accordance with the principles of the Declaration of Helsinki, as revised in 2013.

### 2.2. Steatosis Grading by Liver Histology

After obtaining a cylinder of liver tissue weighing approximately 15 milligrams, we fixed and embedded it in paraffin for histological processing, then cut 4 μM-thick tissue slices with a microtome (Leica Biosystems, Deer Park, IL, USA). We stained tissue slices with hematoxylin–eosin to visualize cell nuclei and cytoplasm, and quantified lobular inflammation as mild, moderate, or severe according to the Brunt scoring system. We graded steatosis in all 38 liver biopsies using the non-alcoholic fatty liver disease activity score (NAS), as follows: No steatosis, grade 0 (<5% hepatocytes containing fat); mild steatosis, grade 1 (5–33% hepatocytes containing fat); moderate steatosis, grade 2 (33–66% hepatocytes containing fat); and severe steatosis, grade 3 (>66% hepatocytes containing fat). We also stained liver slices with Masson’s trichrome to assess fibrosis stage using the Brunt scoring system.

### 2.3. Caveolin 1 Quantification by Western Blot

We also quantified caveolin-1 protein in all liver specimens as an additional analysis to confirm the degree of steatosis. Briefly, we homogenized 30 mg of liver tissue in RIPA lysis buffer supplemented with protease inhibitors (Thermo Fisher Scientific Inc., Waltham, MA, USA). After quantifying protein concentration by Bradford assay, we separated liver lysates by SDS–PAGE and transferred polyacrylamide gels to PVDF membranes. Then, we incubated PVDF membranes with rabbit monoclonal anti–caveolin-1 antibody (1:1000, Cell Signaling Technology, Danvers, MA, USA), followed by an HRP-conjugated secondary antibody (1:5000, Thermo Fisher Scientific Inc., Waltham, MA, USA). We visualized bands by chemiluminescence and normalized using β-actin as a loading control.

### 2.4. Demographic, Anthropometric, Clinical, and Biochemical Parameters

We collected patients’ data from the electronic records of the General Hospital of Mexico. Demographic, anthropometric, and clinical information included sex, age, height, body weight, body mass index (BMI), and the presence of comorbidities, including hypertension, T2D, and chronic kidney disease (CKD). Biochemical variables were fasting blood glucose, triglycerides, total cholesterol, low-density lipoproteins (LDL-C), high-density lipoproteins (HDL-C), alanine aminotransferase (ALT), aspartate aminotransferase (AST), gamma-glutamyl transferase (GGT), alkaline phosphatase (AP), total bilirubin (TB), direct bilirubin (DB), and indirect bilirubin (IB), urea, creatinine, uric acid, hemoglobin, prothrombin time (PT), international normalized ratio (INR), activated partial thromboplastin time (aPTT), and platelet and leukocyte counts. Highly qualified technicians from the Central Laboratory of the General Hospital of Mexico used the Beckman Coulter DxC 700 AU Chemistry Analyzer (Beckman Coulter Inc., Brea, CA, USA) and the BCS^®^ XP System (Siemens Healthcare GmbH, Erlangen, Germany) to measure all laboratory and hematocrit variables.

### 2.5. Measurement of Serum Markers of BBB Dysfunction and LGSI

We collected 6 mL of venous blood from each patient using golden-cap tubes (Vacutainer; BD Diagnostics, Franklin Lakes, NJ, USA). Afterward, we centrifuged blood samples at 1800× *g* for 10 min at room temperature to isolate serum, which was then aliquoted and stored at −70 °C until use. We measured the serum levels of NSE, TGM2, GFAP, TNF-α, and IL-10 using Magnetic Luminex^®^ Performance Assay Kits (R&D Systems, Minneapolis, MN, USA), calculating analyte concentrations in pg/mL or ng/mL based on the corresponding standard curves.

### 2.6. Statistics

We assessed data normality using the Shapiro–Wilk test. We compared numerical variables using Student’s t-test or one-way analysis of variance (ANOVA) with Tukey’s multiple-comparison test, depending on the number of groups. We analyzed the categorical variables using the Chi-squared test. We calculated Pearson correlation coefficients (*r*) and *p*-values to estimate bivariate statistical correlations. We adjusted results for potential confounding variables using multiple regression analysis. We reported results as mean ± standard deviation, absolute values, or percentages, and considered differences significant at *p* < 0.05. We performed statistical analyses using GraphPad Prism 7 and R 3.5.1.

## 3. Results

We enrolled 38 patients with morbid obesity, obtaining a liver biopsy from each to grade steatosis. We found a global prevalence of liver steatosis of 78.9% in our study cohort. There were no differences in sex proportion, age, or BMI among study participants according to steatosis degree; most study subjects were women aged 38.7 ± 9.9 years with an average BMI of 42.3 ± 7.9 kg/m^2^ corresponding to obesity grade 3 or morbid obesity (Table 1). We found no significant differences in the prevalence of comorbidities among patients with no steatosis or with mild, moderate, or severe hepatic fat accumulation, including hypertension, T2D, or CKD (Table 1). Blood levels of glucose, cholesterol, triglycerides, LDL-C, and HDL-C showed no differences among patients with different degrees of steatosis. There were also no differences in kidney function tests, including urea, creatinine, and albumin. Moreover, hemoglobin concentration and thyroid hormone profile did not differ among groups with varying degrees of steatosis. Liver function tests, including AP, TB, DB, IB, AST, ALT, and GGT, showed no differences among different grades of steatosis (Table 1). It is worth noting that IL-10 serum levels decreased by 3-fold in patients with severe steatosis compared to the IL-10 average amount observed in the whole study population. Notably, serum IL-10 was 4-fold less in the severe steatosis group than in patients with mild steatosis (*p* = 0.038) (Table 1). Conversely, serum TNF-α levels increased with the degree of steatosis, with a 2-fold significant rise in patients with severe steatosis compared to participants without steatosis (*p* = 0.029) (Table 1).

**Figure 1:**
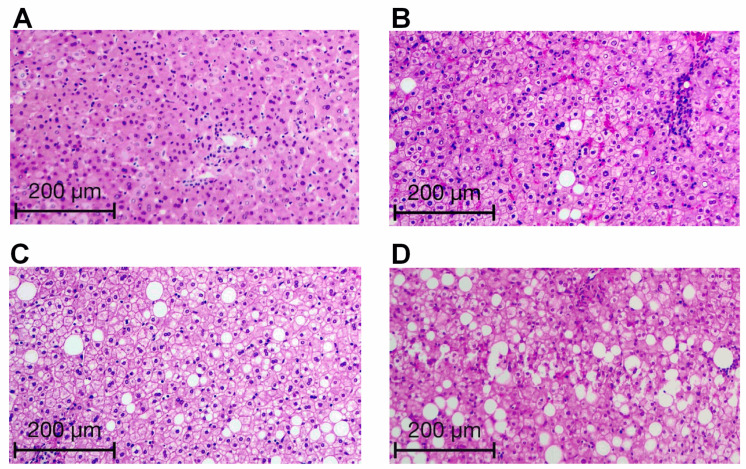
Representative microphotographs of liver tissue in patients with mild, moderate, severe, or no steatosis. (**A**) Liver specimen without steatosis. (**B**) Liver tissue belonging to the mild steatosis group. (**C**) Liver sample belonging to the moderate steatosis group. (**D**) Liver tissue belonging to the severe steatosis group. We stained tissue slices with hematoxylin–eosin and graded lobular inflammation, ballooning, and fibrosis by the Brunt scoring system. We used the NAS to rank steatosis as follows: no steatosis, <5% hepatic fat in liver biopsy; Mild, 5–33% hepatic fat in liver biopsy; Moderate, 33–66% hepatic fat in liver biopsy; Severe, >66% hepatic fat in liver biopsy.

Figure 1 illustrates steatosis grading in the study subjects, ranging from a few lipid droplets in the liver parenchyma to mild, moderate, and abundant steatotic vacuoles. Liver specimens from morbidly obese patients without steatosis showed absence of cytoplasmic vacuoles (*n* = 8) (Figure 1A). Patients with mild steatosis exhibited a few lipid droplet depositions in the liver, compromising just around 15% of hepatocytes (*n* = 17) (Figure 1B). Hepatic tissue from patients with moderate steatosis displayed an increased number of steatotic vacuoles affecting around 50% of hepatocyte cells (*n* = 8) (Figure 1C). Morbidly obese patients within the severe steatosis group showed abundant cytoplasmic vacuoles in the liver parenchyma, compromising more than 70% of hepatocytes (*n* = 5) (Figure 1D).

**Figure 2:**
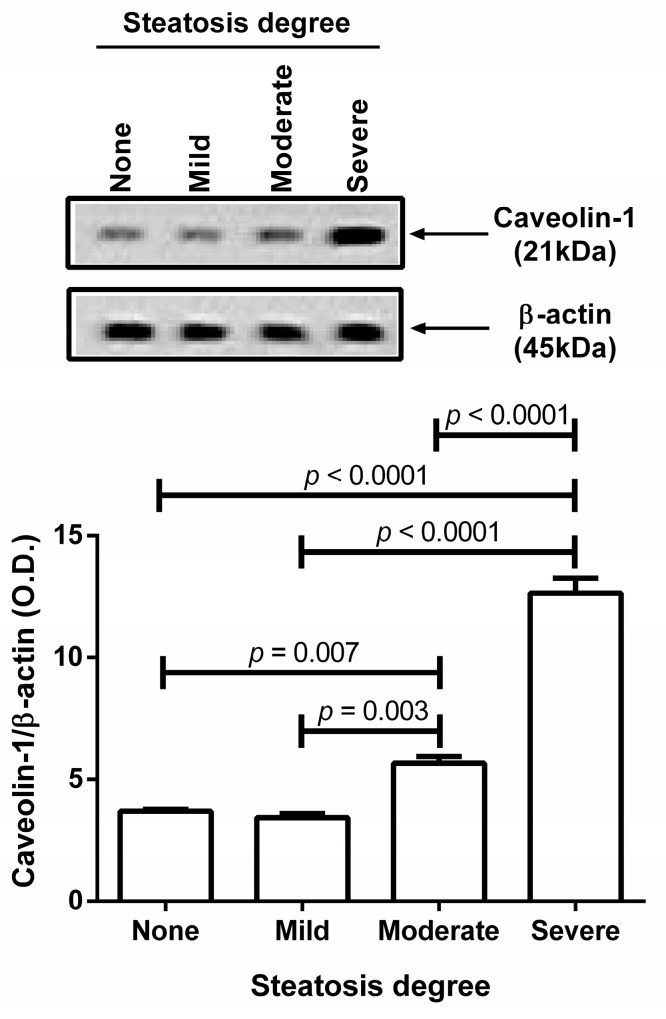
Caveolin-1 protein quantification by Western blot. We measured caveolin-1 protein by Western blot to confirm the degree of steatosis in all liver specimens. Liver samples from patients without steatosis or mild steatosis exhibited reduced caveolin-1 protein production. Liver tissue from patients with moderate steatosis showed a slight increase in caveolin-1 synthesis. Liver specimens from patients with severe steatosis presented a significant increase in caveolin-1 production. We compared differences using one-way ANOVA followed by a post hoc Tukey’s test and reported data as mean ± standard deviation. We considered significant differences when *p* < 0.05. No steatosis, <5% hepatic fat in liver biopsy; Mild, 5–33% hepatic fat in liver biopsy; Moderate, 33–66% hepatic fat in liver biopsy; Severe, >66% hepatic fat in liver biopsy. Abbreviations: O.D., optical density.

Figure 2 depicts caveolin-1 protein quantification by Western blot to confirm the degree of steatosis in all liver specimens. Liver samples from patients without steatosis or mild steatosis exhibited reduced caveolin-1 protein production. In contrast, liver tissue from patients with moderate steatosis showed a slight increase in caveolin-1 synthesis (Figure 2). Liver specimens from patients with severe steatosis presented a significant increase in caveolin-1 production (Figure 2).

Histological characteristics of liver tissue appear in Table 2. The Chi-square test revealed that lobular inflammation and ballooning did not differ among groups with varying degrees of steatosis (Table 2). However, liver fibrosis was significantly more frequent in patients with moderate or severe steatosis than in those with mild steatosis, as assessed by the Brunt scoring system (Table 2). Furthermore, liver biopsies from patients with severe steatosis tended to exhibit significantly higher NAS than those registered in patients without steatosis (Table 2).

**Figure 3:**
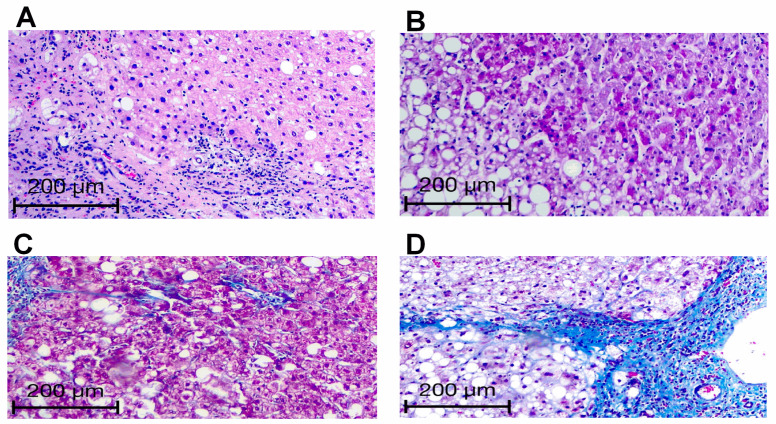
Representative microphotographs of Masson’s trichrome stain in liver tissue of patients with mild, moderate, severe, or no steatosis. The microphotographs show Masson’s trichrome staining to extend visualization of fibrosis with increasing steatosis severity. Liver specimens from patients without steatosis or mild steatosis exhibited no collagen fiber deposition (**A** and **B**, respectively). Liver tissue from patients with moderate steatosis showed a slight increase in collagen-positive staining, mainly around the portal veins (**C**). Liver samples from patients with severe steatosis showed more often significant collagen depositions forming septa around periportal areas (**D**). We graded fibrosis by the Brunt scoring system.

Figure 3 provides Masson’s trichrome stains to extend the visualization of fibrosis by steatosis severity. Liver specimens from patients without steatosis or mild steatosis exhibited no collagen fiber deposition (Figure 3A and Figure 3B, respectively). Liver tissue from patients with moderate steatosis had a slight increase in collagen-positive stain, mainly around the portal veins (Figure 3C). Conversely, patients with severe steatosis showed more often significant collagen depositions forming septa around periportal areas (Figure 3D).

**Figure 4:**
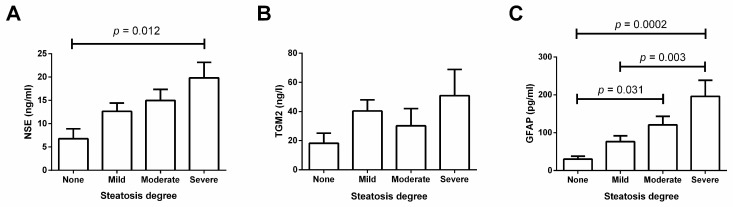
Serum levels of NSE, TGM2, and GFAP in patients with mild, moderate, severe, or no steatosis. (**A**) NSE serum levels gradually increased with increasing steatosis severity. (**B**) TGM2 circulating levels did not change among patients with varying degrees of steatosis. (**C**) GFAP serum levels increased in proportion to steatosis severity. We compared differences using one-way ANOVA followed by a post hoc Tukey’s test and reported data as mean ± standard deviation. We considered significant differences when *p* < 0.05. No steatosis, <5% hepatic fat in liver biopsy; Mild, 5–33% hepatic fat in liver biopsy; Moderate, 33–66% hepatic fat in liver biopsy; Severe, >66% hepatic fat in liver biopsy. Abbreviations: NSE, neuron-specific enolase; TGM2, transglutaminase 2; GFAP, glial fibrillary acidic protein.

Figure 4 illustrates serum levels of markers of BBB dysfunction in patients with different grades of steatosis. NSE serum levels gradually increased with increasing steatosis severity, with a significant 3-fold elevation in patients with severe steatosis compared to participants without steatosis (*p* = 0.012) (Figure 4A). TGM2 serum levels tended to increase as steatosis also rose; however, there were no significant differences (Figure 4B). Similarly to NSE, GFAP serum levels increased in the same proportion as steatosis severity, showing significant differences between patients with severe steatosis and those with mild steatosis or absence of steatosis (*p* = 0.003 and *p* = 0.0002, respectively) (Figure 4C). Patients with moderate steatosis also exhibited a significant 4-fold increase in GFAP serum levels compared to patients without steatosis (*p* = 0.031) (Figure 4C). It is worth noting that differences in cytokine serum levels and circulating markers of BBB disruption were unaltered after adjusting for potentially confounding variables, including sex, age, and comorbidities.

**Figure 5:**
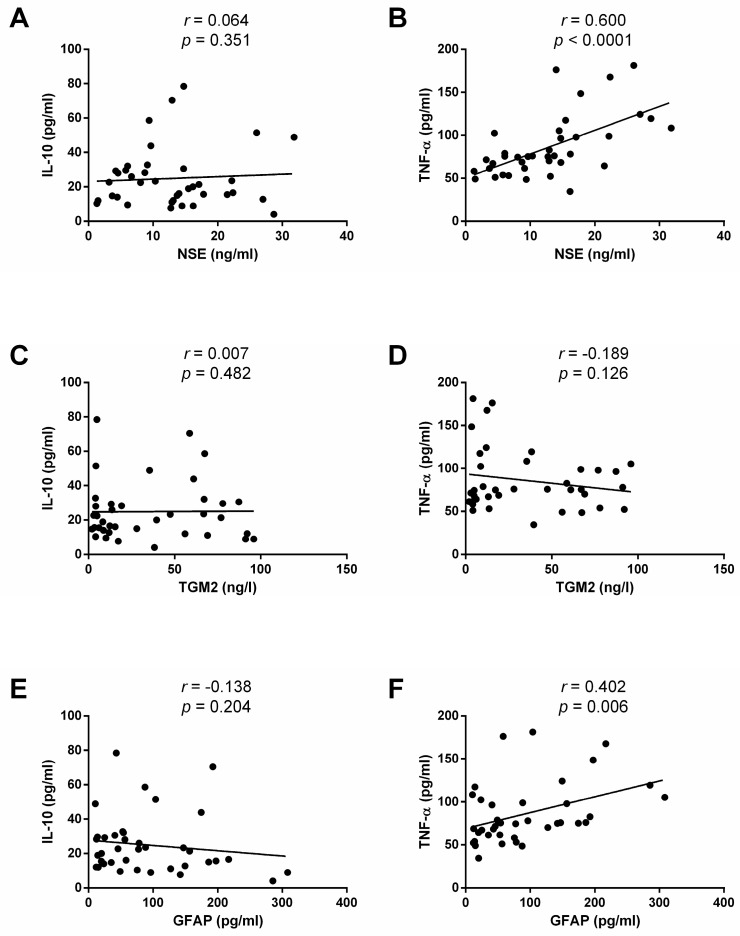
Correlation analyses for IL-10 and TNF-α with NSE, TGM2, and GFAP in patients with mild, moderate, severe, or no steatosis. (**A**) IL-10 circulating levels showed no significant association with NSE serum levels. (**B**) TNF-α exhibited a significantly positive correlation with NSE. (**C**) IL-10 did not have a significant association with TGM2. (**D**) TNF-α was not associated with TGM2 serum levels. (**E**) IL-10 showed no significant correlation with GFAP. (**F**) TNF-α circulating levels exhibited a significantly positive association with GFAP serum levels. We calculated the coefficients (*r*) and *p*-values using Pearson’s correlation analysis. We considered significant differences when *p* < 0.05. No steatosis, <5% hepatic fat in liver biopsy; Mild, 5–33% hepatic fat in liver biopsy; Moderate, 33–66% hepatic fat in liver biopsy; Severe, >66% hepatic fat in liver biopsy. Abbreviations: IL-10, interleukin-10; TNF-α, tumor necrosis factor-alpha; NSE, neuron-specific enolase; TGM2, transglutaminase 2; GFAP, glial fibrillary acidic protein.

The serum levels of markers of BBB alteration increased not only with steatosis severity but also were associated with systemic inflammatory mediators. Serum NSE showed no correlation with IL-10 circulating levels (Figure 5A). However, NSE exhibited a positive, significant association with TNF-α serum levels, increasing in the same proportion as this proinflammatory circulating marker (*r* = 0.600, *p* < 0.0001) (Figure 5B). TGM2 did not have any correlations with IL-10 or TNF-α serum levels (Figure 5C and Figure 5D, respectively). GFAP circulating levels were not associated with serum IL-10 (Figure 5E). Conversely, there was a significant, positive correlation between GFAP and TNF-α; in fact, GFAP increased proportionally to TNF-α (*r* = 0.402, *p* < 0.006) (Figure 5F).

## 4. Discussion

The present study demonstrates that the degree of liver steatosis is associated with higher circulating levels of markers of BBB disruption and systemic inflammation, particularly NSE, GFAP, and TNF-α. It is noteworthy that elevated serum levels of markers of BBB dysfunction occur in the absence of altered liver function tests, metabolic parameters, or comorbidities, suggesting an association between BBB dysfunction and the development of steatosis.

Our findings align with previous evidence showing that BBB impairment is associated with the occurrence of metabolic disease and systemic inflammation. A clinical study demonstrated that one in five patients with AD has severe insulin resistance, accompanied by an elevation in the albumin cerebrospinal fluid/serum index, suggesting loss of BBB integrity parallel to disturbances in insulin sensitivity [26]. Likewise, an experimental study reported that mice with high-fat diet-induced metabolic syndrome exhibit BBB vascular leakage, associated with loss of tight junction proteins and the basal lamina of endothelial cells and astrocytes [27]. In parallel, this study reported that BBB disruption also led to enhanced migration of CD45 + CD3 + immune cells into the brain parenchyma and increased TNF-α production, supporting a link among BBB, metabolic syndrome, and systemic inflammation [27]. As is well known, steatosis is a hepatic manifestation of insulin resistance, metabolic syndrome, and systemic inflammation, which opens an interesting avenue for exploring the possible role of BBB dysfunction in its pathogenesis [28,29]. In this context, our results showing increased serum levels of TNF-α, NSE, and GFAP expand the body of evidence suggesting an association among BBB disruption, systemic inflammation, and liver steatosis.

Several biological mechanisms may help to explain the probable relationship among BBB disruption, systemic inflammation, and liver steatosis. First, accumulating evidence shows that TNF-α can induce endothelial cell apoptosis and inhibit claudin and occludin synthesis, thereby altering tight junction structure and BBB permeability. In this regard, a prior study reported that TNF-α promotes apoptosis in human umbilical vascular endothelial cells (HUVECs) by inhibiting Bcl-2 and activating Caspase-3 [30]. Additionally, in vitro exposure of LLC-PK1 tubular epithelial cells to TNF-α decreases claudin-2 expression, a key member of the claudin family proteins that form tight junctions in the BBB and other barrier structures [31]. Similarly, treatment of human cerebral microvascular endothelial cells (hCMECs) with TNF-α reduces occludin synthesis via hypoxia-inducible factor 1 alpha (HIF-1α), another crucial component of tight junctions in the BBB [32]. In line with our results, numerous reports indicate that circulating TNF-α levels increase in morbid obesity and MASLD [33,34,35]. In this scenario, TNF-α may down-regulate both cellular and molecular components of the BBB, thereby facilitating the leakage of centrally produced molecules into the circulation, such as NSE and GFAP. Our findings show significant correlations between serum TNF-α and circulating NSE and GFAP levels, supporting the idea that this proinflammatory cytokine may interfere with BBB structure and contribute to the development of steatosis.

Second, LGSI can promote endothelial dysfunction in both hepatic sinusoids and cerebral microvasculature via proinflammatory factors such as TNF-α and nitric oxide [36,37]. Thus, systemic inflammation may lead to parallel endothelial injury in both organs, causing liver endotoxemia and steatosis, as well as BBB damage, evidenced by the leakage of molecules such as NSE and GFAP. Third, the low serum levels of IL-10 observed in morbidly obese patients with severe steatosis may exacerbate systemic inflammation, thus amplifying the possible effects of TNF-α on the BBB. IL-10 is a cytokine with potent anti-inflammatory actions that counteract TNF-α’s proinflammatory effects and limit LGSI [38,39]. IL-10 serum levels decrease in morbid obesity, metabolic dysfunction, and MASLD, all of which are featured by systemic inflammation [40,41,42]. In line with this information, we found that serum IL-10 levels are lowest in patients with severe steatosis, wherein we also observed the highest TNF-α, NSE, and GFAP levels. The levels and correlations among IL-10, TNF-α, NSE, and GFAP observed in our study suggest that BBB dysfunction may be associated with systemic inflammation and contribute to the development of severe steatosis. Although plausible, we must acknowledge that the cross-sectional nature of the study does not allow us to establish a causal role of BBB disruption in the development of liver steatosis.

It is worth noting that increased serum levels of NSE and GFAP do not necessarily indicate that the BBB directly contributes to liver steatosis. In fact, systemic inflammation could also promote hepatic lipid accumulation and show only a mere association with indirect markers of BBB disruption. In this regard, a prior study in a mouse model demonstrated that Kupffer cells can overproduce TNF-α, which, in turn, can suppress fatty acid oxidation and lead to lipid accumulation in liver cells [43,44]. Notably, indirect serum markers of neuroinflammation and BBB dysfunction, such as NSE, are elevated in patients with metabolic syndrome and neurodegenerative disorders, both characterized by high circulating TNF-α levels [45,46]. Therefore, we must consider other potential actors in the pathogenesis of liver steatosis beyond the BBB, such as systemic inflammation.

Present findings support a link between liver steatosis and BBB alterations in the context of morbid obesity. The progressive elevation in NSE and GFAP alongside steatosis severity and LGSI markers suggests that hepatic lipid accumulation is not an isolated metabolic event but part of a systemic, multi-organ process involving the BBB, liver, and immune system. As mentioned above, TNF-α may impair tight junction integrity and increase BBB permeability, facilitating the leakage of NSE and GFAP into the systemic circulation [47,48]. In turn, BBB disruption may exacerbate TNF-α production by promoting immune cell trafficking into the CNS, thereby amplifying neuroinflammatory and systemic inflammatory responses in a bidirectional feedback loop. Although metabolic parameters did not differ across steatosis grades in this study, the association between hepatic lipid accumulation and fibrosis in patients with severe steatosis supports an association of BBB and LGSI with liver disease [49,50]. These findings collectively align with emerging evidence showing increased steatosis prevalence in patients with neurological disorders, a scenario where the BBB may act as a mediator connecting cerebral and liver pathology [14,15].

Although our work provides a solid link between BBB dysfunction and systemic inflammation in the pathogenesis of liver steatosis, we must acknowledge that the study has some limitations. First, the relatively small sample size is an important limitation that may reduce the generalizability of our findings to broader populations, particularly when performing subgroup analyses by degree of steatosis. This study’s limitation may affect the statistical power to detect slight but significant differences among subgroups. We decided to grade steatosis by liver biopsy, which impacted the number of patients enrolled in the study but improved our ability to establish strong correlations among steatosis degree, systemic inflammatory markers, and BBB impairment. Second, the cross-sectional study design does not allow us to know whether BBB disruption directly contributes to steatosis development or is merely associated with systemic inflammation and metabolic dysfunction. Third, we indirectly estimated BBB integrity by examining blood levels of NSE, TGM2, and GFAP rather than using neuroimaging or functional studies such as quantitative positron emission tomography (qPET). Fourth, altered serum levels of NSE and GFAP may reflect downstream effects of neuroinflammation rather than direct BBB disruption; thus, we cannot establish a causal role for BBB integrity in liver steatosis. Finally, we must acknowledge that the statistical correlations we observed between markers of BBB disruption and circulating cytokines were weak to moderate, so we advise caution when interpreting the resulting associations.

## 5. Conclusions

Our findings indicate that steatosis severity in patients with morbid obesity is significantly associated with increased serum levels of markers of BBB disruption and systemic inflammation, including NSE, GFAP, and TNF-α. The highest degree of steatosis is also associated with a significant increase in TNF-α and a clear decrease in IL-10, which, in turn, correlates with NSE and GFAP serum levels, thereby suggesting that systemic inflammation accompanies BBB dysfunction and steatosis. These data highlight the potential role of the BBB in the liver–brain axis, opening exciting avenues to explore how BBB disruption may contribute to hepatic lipid accumulation.

## Figures and Tables

**Table 1:** Demographic, clinical, and laboratory parameters of the study population.

		Steatosis Degree				
Variables	All Patients	None ^a^	Mild ^b^	Moderate ^c^	Severe ^d^	*p*-Value
Sex proportion (w/m)	31/7	7/1	14/3	6/2	3/2	0.653
Age (years)	38.7 ± 9.9	40.0 ± 10.8	35.4 ± 8.3	43.2 ± 8.8	44.2 ± 11.3	0.089
BMI (kg/m^2^)	42.3 ± 7.9	41.1 ± 4.0	43.1 ± 11.2	42.6 ± 3.6	41.3 ± 4.3	0.136
Hypertension (%)	34.2	37.5	35.2	25.0	40.0	0.604
Type 2 diabetes (%)	28.9	25.0	29.4	37.5	20.0	0.909
CKD (%)	21.0	12.5	23.5	25.0	20.0	0.920
Glucose (mg/dL)	128.7 ± 46.9	119.8 ± 48.6	132.9 ± 48.5	142.5 ± 52.8	106.2 ± 25.8	0.535
Total cholesterol (mg/dL)	181.8 ± 48.9	193.1 ± 48.6	162.1 ± 34.9	200.0 ± 65.6	201.8 ± 50.4	0.162
Triglycerides (mg/dL)	167.8 ± 43.7	176.5 ± 29.4	167.6 ± 58.3	164.0 ± 34.7	160.6 ± 13.2	0.922
LDL-C (mg/dL)	129.2 ± 25.1	128.1 ± 17.0	131.9 ± 32.2	127.9 ± 19.2	123.6 ± 20.7	0.927
HDL-C (mg/dL)	38.7 ± 8.7	38.6 ± 9.4	36.5 ± 7.6	41.2 ± 9.6	42.4 ± 9.8	0.461
Urea (mg/dL)	12.3 ± 7.2	26.8 ± 13.1	31.0 ± 9.6	34.4 ± 13.1	33.9 ± 14.2	0.582
Creatinine (mg/dL)	0.7 ± 0.1	0.7 ± 0.1	0.7 ± 0.1	0.7 ± 0.1	0.8 ± 0.08	0.536
Uric acid (mg/dL)	7.6 ± 1.6	7.1 ± 1.3	7.3 ± 1.6	8.7 ± 1.3	7.7 ± 1.6	0.132
Hemoglobin (mg/dL)	13.3 ± 2.0	13.6 ± 1.1	13.3 ± 1.8	12.6 ± 3.1	14.1 ± 1.7	0.615
TSH (mU/L)	2.9 ± 1.7	2.6 ± 1.7	3.2 ± 1.9	2.8 ± 1.4	2.5 ± 2.0	0.813
FT3 (mU/L)	2.6 ± 0.7	2.7 ± 0.4	2.4 ± 0.8	2.7 ± 0.9	3.0 ± 0.4	0.332
FT4 (mU/L)	0.7 ± 0.1	0.7 ± 0.1	0.7 ± 0.1	0.7 ± 0.1	0.7 ± 0.1	0.817
AP (IU/L)	109.0 ± 22.0	100.1 ± 21.5	105.4 ± 23.1	125.3 ± 19.8	109.4 ± 9.9	0.100
Albumin (g/L)	33.2 ± 2.7	33.5 ± 2.8	33.8 ± 2.5	31.1 ± 2.8	34.6 ± 2.1	0.077
TB (mg/dL)	0.5 ± 0.1	0.5 ± 0.03	0.5 ± 0.1	0.5 ± 0.1	0.6 ± 0.2	0.198
DB (mg/dL)	0.2 ± 0.09	0.2 ± 0.1	0.2 ± 0.08	0.1 ± 0.08	0.2 ± 0.09	0.168
IB (mg/dL)	0.3 ± 0.1	0.2 ± 0.1	0.3 ± 0.1	0.3 ± 0.1	0.4 ± 0.2	0.226
AST (IU/L)	30.1 ± 6.2	29.3 ± 8.1	30.0 ± 6.1	31.5 ± 4.9	29.2 ± 6.7	0.901
ALT (IU/L)	36.8 ± 7.0	40.1 ± 4.7	34.6 ± 8.1	36.2 ± 7.3	40.2 ± 2.2	0.211
GGT (IU/L)	39.4 ± 10.1	38.0 ± 10.9	42.1 ± 10.1	38.5 ± 10.0	33.8 ± 8.2	0.400
IL-10 (pg/mL)	24.9 ± 17.2	19.5 ± 7.0	31.7 ± 21.0	25.9 ± 12.7	8.4 ± 3.1	^b^_vs_^d^ 0.038 *
TNF-α (pg/mL)	88.6 ± 38.5	66.0 ± 24.0	81.4 ± 30.5	106.8 ± 51.0	120.4 ± 35.4	^a^_vs_^d^ 0.029 *

We compared differences using one-way ANOVA followed by a post hoc Tukey’s test, except for sex proportion, which was analyzed using Chi-square test. We reported data as mean ± standard deviation, percentages, or absolute numbers, and used asterisks to indicate significant differences when *p* < 0.05. Letter a represents patients with no steatosis (none), while b, c, and d represent patients in the mild, moderate, or severe steatosis groups, respectively. No steatosis, <5% hepatic fat in liver biopsy; Mild, 5–33% hepatic fat in liver biopsy; Moderate, 33–66% hepatic fat in liver biopsy; Severe, >66% hepatic fat in liver biopsy. Abbreviations: w, women; m, men; BMI, body mass index; LDL-C, low-density lipoprotein cholesterol; HDL-C, high-density lipoprotein cholesterol; TSH, thyroid-stimulating hormone; FT3, free-triiodothyronine; FT4, free-thyroxine; AP, alkaline phosphatase; TB, total bilirubin; IB, indirect bilirubin; DB, direct bilirubin; ALT, alanine aminotransferase; AST, aspartate aminotransferase; GGT, gamma-glutamyl transferase; IL-10, interleukin-10; TNF-α, tumor necrosis factor-alpha.

**Table 2:** Liver histology characteristics for patients with mild, moderate, severe, or no steatosis.

		Frequency According to Steatosis Degree				
Liver Histology	Score	None	Mild	Moderate	Severe	*p*-Value
Lobular inflammation	0	2	3	0	0	0.076
	1	5	10	1	2	
	2	1	4	6	2	
	3	0	0	1	1	
Ballooning	0	1	0	0	0	0.123
	1	6	15	5	2	
	2	1	2	3	3	
Fibrosis	0	4	10	0	0	0.015 *
	1	3	6	4	2	
	2	1	1	4	2	
	3	0	0	0	1	
NAS	0	1	0	0	0	<0.001 *
	1	1	0	0	0	
	2	4	3	0	0	
	3	2	8	0	0	
	4	0	6	1	0	
	5	0	0	3	2	
	6	0	0	4	1	
	7	0	0	0	2	

We compared scores with the Chi-square test. We reported data as absolute numbers and used asterisks to indicate significant differences when *p* < 0.05. No steatosis, <5% hepatic fat in liver biopsy; Mild, 5–33% hepatic fat in liver biopsy; Moderate, 33–66% hepatic fat in liver biopsy; Severe, >66% hepatic fat in liver biopsy. We graded lobular inflammation, ballooning, and fibrosis using the Brunt scoring system, and ranked steatosis using the NAS. Abbreviations: NAS, non-alcoholic fatty liver disease activity score.

## Data Availability

Data are available upon request.
